# Prior respiratory syncytial virus infection reduces vaccine-mediated Th2-skewed immunity, but retains enhanced RSV F-specific CD8 T cell responses elicited by a Th1-skewing vaccine formulation

**DOI:** 10.3389/fimmu.2022.1025341

**Published:** 2022-10-04

**Authors:** Katherine M. Eichinger, Jessica L. Kosanovich, Timothy N. Perkins, Tim D. Oury, Nikolai Petrovsky, Christopher P. Marshall, Mark A. Yondola, Kerry M. Empey

**Affiliations:** ^1^ Department of Pharmaceutical Sciences, University of Pittsburgh School of Pharmacy, University of Pittsburgh, Pittsburgh, PA, United States; ^2^ Department of Pathology, University of Pittsburgh School of Medicine, University of Pittsburg, Pittsburgh, PA, United States; ^3^ Vaxine Pty Ltd., Bedford Park, SA, Australia; ^4^ College of Medicine and Public Health, Flinders University, Bedford Park, SA, Australia; ^5^ Calder Biosciences, New York City, NY, United States; ^6^ Department of Pharmacy and Therapeutics, University of Pittsburgh School of Pharmacy, University of Pittsburgh, Pittsburgh, PA, United States; ^7^ Center for Clinical Pharmaceutical Sciences, University of Pittsburgh School of Pharmacy, University of Pittsburgh, Pittsburgh, PA, United States; ^8^ Department of Immunology, University of Pittsburgh School of Medicine, University of Pittsburgh, Pittsburgh, PA, United States

**Keywords:** RSV, vaccine enhanced disease, mice, Th2, seropositive

## Abstract

Respiratory syncytial virus (RSV) remains the most common cause of lower respiratory tract infections in children worldwide. Development of a vaccine has been hindered due the risk of enhanced respiratory disease (ERD) following natural RSV exposure and the young age (<6 months) at which children would require protection. Risk factors linked to the development of ERD include poorly neutralizing antibody, seronegative status (never been exposed to RSV), and a Th2-type immune response. Stabilization of the more antigenic prefusion F protein (PreF) has reinvigorated hope for a protective RSV vaccine that elicits potent neutralizing antibody. While anecdotal evidence suggests that children and adults previously exposed to RSV (seropositive) are not at risk for developing vaccine associated ERD, differences in host immune responses in seropositive and seronegative individuals that may protect against ERD remain unclear. It is also unclear if vaccine formulations that skew towards Th1- versus Th2-type immune responses increase pathology or provide greater protection in seropositive individuals. Therefore, the goal of this work was to compare the host immune response to a stabilized prefusion RSV antigen formulated alone or with Th1 or Th2 skewing adjuvants in seronegative and seropositive BALB/c mice. We have developed a novel BALB/c mouse model whereby mice are first infected with RSV (seropositive) and then vaccinated during pregnancy to recapitulate maternal immunization strategies. Results of these studies show that prior RSV infection mitigates vaccine-mediated skewing by Th1- and Th2-polarizing adjuvants that was observed in seronegative animals. Moreover, vaccination with PreF plus the Th1-skewing adjuvant, Advax, increased RSV F85-93-specific CD8 T cells in both seronegative and seropositive dams. These data demonstrate the importance of utilizing seropositive animals in preclinical vaccine studies to assess both the safety and efficacy of candidate RSV vaccines.

## Introduction

Respiratory syncytial virus (RSV) is the leading viral cause of lower respiratory tract infections in infants and children worldwide. Severe RSV disease peaks at 2 months of age (1) and children less than 6 months of age account for approximately 50% of all RSV-related hospital admissions and in-hospital deaths (2). The young age at which RSV disease is most severe highlights the need for robust protection early after birth. However, significant safety concerns surrounding direct infant RSV vaccination came to light after a 1966 formalin-inactivated RSV vaccine (FIRSV) trial, whereby vaccinated children developed an enhanced respiratory disease (ERD) following natural RSV infection that was not observed in the control group that received parainfluenza vaccine (3). Results from the FIRSV studies and subsequent clinical studies have reported no cases of vaccine associated ERD among children or adults that were RSV seropositive prior to vaccination (3–7). It is postulated that prior RSV infection protects against vaccine associated ERD, though the immune environment under which such protection is imparted remains unclear.

Because many young children at risk for severe disease have not been previously infected with RSV, direct infant vaccination continues to pose a risk for developing ERD. To mitigate such a potential risk, maternal immunization strategies have garnered significant enthusiasm as an alternative means to provide RSV-specific neutralizing antibodies to children immediately after birth. Maternal immunization is believed to remove the risk of eliciting CD4 Th2 inflammation thought to be associated with direct-infant vaccine associated ERD. The risk of vaccine associated ERD among pregnant mothers is presumed to be low, as it is likely they have been previously exposed to RSV throughout their lifetime. However, the full benefits and risks of maternal vaccination to the mother, remains unclear. Despite several completed and ongoing clinical studies to assess maternal immunization strategies, little data is available on viral protection in the immunized mothers. However, recently published findings from a phase II study to assess the safety and immunogenicity of an RSV F protein nanoparticle vaccine in pregnant women reported no differences in acute respiratory infections between placebo and immunized mothers (8).

Thus, the goals of the current study were to determine the extent to which immunization of seropositive (previously infected with RSV) versus seronegative (RSV naïve) BALB/cJ dams protected against RSV infection and to define differential immune responses in each cohort based on serostatus. Sero+ or sero- BALB/cJ dams were immunized with a stabilized prefusion RSV F protein (9), shown to induce high levels of neutralizing antibody in cotton rats and BALB/c mice. As vaccine associated-ERD is heavily linked to Th2-mediated immune pathology (10), dams within each sero+ and sero- group were immunized with PreF alone (PreF) or formulated with the Th2 adjuvant, alum (PreFAlum) – shown to stimulate CD4 Th2 immune cells (11), or with the Th1-skewed adjuvant, Advax (PreFAdx) (12–17). Viral protection, lung pathology, and immune responses were compared based on serostatus of the animals prior to immunization and the vaccine regimen they received relative to those that received vehicle only.

Results of these studies demonstrated that immunization of sero- dams with PreF and PreFAlum markedly increased type II cytokine production by CD4 T cells and ILC2s, in concert with extensive mucus production and eosinophilic lung inflammation, which was completely mitigated in animals immunized with PreFAdx. Conversely, while prior RSV exposure resulted in a faster or more robust immune response overall, type II immunity and lung inflammation were not increased in the PreF and PreFAlum groups compared to vehicle controls and only the PreFAlum group demonstrated a significant increase in mucus production. However, animals immunized with the Th1-skewing vaccine formulation, PreFAdvax, demonstrated increased RSV F_85-93_-specific CD8 T cells in both sero- and sero+ dams. Taken together, these results highlight the importance of incorporating seropositive animals in preclinical RSV vaccine studies.

## Materials and methods

### Mice, immunization, and RSV virus

Balb/c female mice were purchased from The Jackson Laboratory (Bar Harbor, ME). The sero+ females were purchased at 4 weeks of age and intranasally (i.n.) challenged with 2.5 x 10^5^ plaque forming units (pfu) of RSV Line 19 (RSV L19; a gift from Martin Moore, Vanderbilt University – now at Meissa Vaccines, CA) per gram of body weight under isoflurane anesthesia as previously described (18). Sero+ females were allowed to recover for 3 weeks prior to immunization. Age-matched females were purchased from The Jackson Laboratory to serve as the sero- cohort. One week prior to breeding, sero+ and sero- females were immunized *via* intramuscular (i.m.) injection with 50mcl of vehicle (Veh.; PBS), RSV Pre-F stabilized with di-tyrosine crosslinks (PreF; Calder Biosciences, NY, NY) alone or formulated with Advax-SM™ (PreFAdx; 1 mg/mouse; Vaxine Pty Ltd, Bedford Park, Australia) or Alum (PreFAlum; 10 mg/mL, Alhydrogel, *In vivo*Gen). The immunized female mice were paired with naïve, unimmunized male breeders one week after prime and dams were boosted three weeks later with their respective vaccine formulations. At 8 weeks post-prime, dams were challenged i.n. with RSV L19 (5 x 10^5^ pfu/gm) and culled at 4 days post-infection (DPI) using 100% isoflurane and cervical dislocation. RSV L19 was propagated and viral titers quantified as previously described (19).

### Cell preparation, cytokine analysis, and flow cytometry

Bronchoalveolar lavage (BAL) was collected through intratracheal instillation of HBSS + EDTA (30 μM). BAL samples were centrifuged, and the soluble fraction stored at -80°C for cytokine analysis and the cellular fraction analyzed *via* flow cytometry. Cytokine concentrations were determined using the Bio-Plex Pro™ Mouse Cytokine 23-plex Assay (BioRad, CA), per manufacturer’s protocol. The right lung was harvested and enzyme-digested into a single cell suspension for flow cytometry, as described previously (20).

BAL cells and lung homogenate were stimulated ex-vivo for intracellular cytokine detection. Briefly, BAL cells were incubated for 3 hours in 10% RPMI supplemented with Brefeldin A (1:1000, eBioscience) prior to surface and intracellular flow staining. Live cells from lung homogenate were stained with trypan blue and enumerated using a hemacytometer, then T cells and type 2 innate lymphoid cells (ILC2) cells were stimulated for cytokine production.

For T cell intracellular cytokine staining, lung homogenate (1 million cells in duplicate) was plated in a CD3-coated (5 mcg/mL, Biolegend) 96-well flat-bottomed tissue culture plate in 200mcl of 10% RPMI supplemented with CD28 (2mcg/mL) and incubated at 37°C overnight. After overnight stimulation with CD3/CD28, lung homogenate underwent a secondary stimulation with PMA (1:1000), ionomycin (1:1000), and Brefeldin A (1:1000) for 2 hours prior to T cell surface and intracellular flow staining. To obtain intracellular ILC2 cytokine staining, lung homogenate (3 million cells) was plated in 24-well tissue culture plates and stimulated with PMA (30 ng/mL), ionomycin (500 ng/mL), and Brefeldin A (1:1000) in 10% RPMI at 37°C for 3 hours.

BAL cells were surface stained with combinations of the following antibodies: CD16/32-2.4G2, Siglec-F-E50-2440, F4/80-T45-2342, CD11b-M1/70, CD4-GK1.5, CD8α-53-6.7 (BD Biosciences, CA), CD11c-N418, CD19-6D5, and TCRβ-H57-597 (Biolegend, CA). Lung homogenate was surface stained with combinations of the following antibodies: CD16/32-2.4G2, Lineage cocktail, CD45-30-F11, ST2-DIH9, IL-7Rα-A7R32, CD19-6D5, TCRβ-H57-597 (Biolegend), CD4-GK1.5, and CD8α-53-6.7 (BD Bioscience). Following surface staining, cells were fixed and permeabilized for intracellular staining with BD CytoFix/CytoPerm™ Solution Kit (BD Biosciences) according to the manufacturer’s protocol. Intracellular cytokines were stained with a combination of the following antibodies: IL-5-TRFK5, IFNγ-XMG1.2 (Biolegend), IL-13-ebio13A, and IL-6-MP5-20F3 (ThermoFisher Scientific, MA). Where indicated and prior to surface staining, BAL samples were incubated with RSV A Strain F-protein _85-93_ MHC I pentamer (H-2kd KYKNAVTEL; Proimmune, FL) to identify RSV F_85-93_ -specific CD8^+^ T cells. Samples were run on a BD LSRFortessa managed by the United Flow Core of the University of Pittsburgh. Data was analyzed using FlowJo V10 software (FLOWJO, LLC, OR).

### Neutralizing antibody - renilla luciferase RSV reporter assay

Pre-challenge serum was collected *via* submandibular bleed 2-3 days prior to RSV challenge and separated using Gel-Z Serum Separator Tubes (Sarstedt, Germany). Serum was stored at -80°C until heat inactivation (56°C for 30 minutes) and neutralizing antibody titers were performed. Serial dilutions of heat inactivated serum (50 mcL in phenol-free MEM supplemented with 5% FBS and Pen/Strep, Invitrogen) were incubated for 2 hours in a 37°C CO_2_ incubator in a 96-well plate format with 100 pfu/well Line 19 RSV-Renilla Luciferase virus (provided by the Moore laboratory) in 50 mcL phenol free MEM medium as above. After 2 hours, Hep-2 cells were trypsinized and a total of 2.5 x 10^4^ cells were added per well in 25 mcL of phenol free MEM with FBS and antibiotics as above. Cells were incubated for a total of 64-66 hours at 37°C at 5% CO_2_ and the luciferase readout was then obtained using the Renilla-glo luciferase kit (Promega) according to the manufacturer’s instructions. Luciferase activity (luminescence) was measured using a Novostar plate reader after a 15-minute incubation at 25°C. All plates were run in duplicate and averaged.

### PreF specific IgG subtype assay

Co-star 96-well, high binding ELISA plates were coated with RSV PreF at a concentration of 5 mcg/ml overnight at 4°C. Each plate included standards of either mouse IgG1 or IgG2a (Invitrogen) at 10 mcg/mL and 2mcg/mL in a 2-fold dilution series for intra-plate quantification of signal on uncoated wells. Plates were then washed with PBS and blocked for 1 hour at 37°C with 1% BSA in PBS. Heat inactivated serum samples were diluted 1:500 in 1% BSA in PBS for the first well, and then 3-fold serially diluted a total of 3 times. Serum was incubated on the plates for 1 hour at 25°C, followed by 3 washes with PBS 0.05% Tween-20, and secondary antibody incubation with anti-IgG1 or anti-IgG2a (isotype specific, BD Pharmingen), respectively at a 1:10,000 dilution for 30 minutes at 25°C in 1% BSA. 1-step TMB (Thermo Scientific) was used to develop the plates and the reaction was quenched by the addition of 4N H_2_SO_4_. Plates were read at 450 nm in a Novostar plate reader. Data analysis was performed in Excel and data points were interpolated from the linear region of the standards on each individual plate. Samples were run in duplicate, and the data presented represents the average values from both runs.

### Histology

Right lungs were gravity filled with 10% formalin at 4 days post RSV challenge, as previously described (21). The McGowan Institute for Regenerative Medicine (University of Pittsburgh, PA) stained and processed the preserved lungs. Periodic Acid-Schiff (PAS) stain was used by two individuals blinded to treatment groups to quantify airway mucus production, according to previously published methods (18). Briefly, a score of 0 - 5 was given to all airways (average 55) with the following scale: 0 = no PAS+ cells; 1 = 1-25% PAS+ cells; 2 = 26-50% PAS+ cells; 3 = 51-75% PAS+ cells; 4 = 76-100% PAS+ cells. Scores were averaged and the total percentage of PAS+ airways were graphed along with a more detailed breakdown of the ratio of each severity score (0 – 5) divided by the total number of airways. Standard hematoxylin and eosin staining was performed on lung sections and scored by two pathologists (Oury and Perkins) blinded to treatment groups, according to previously described methods (22). In short, each field (average 28 fields) in the lung was observed with a light microscope (x200 magnification) and scoring was based on the percentage of lung tissue affected according to the following scale: 0 = no inflammation, 1 = up to 25%, 2 = 25 – 50%, 3 = 50 – 75%, and 4 = 75 – 100%. Scores were averaged and reported as a ratio of the sum of scores divided by the total number of fields counted. Severity scores were also graphed as a fraction of the total number of fields counted.

### Statistical analysis

Statistics were performed with GraphPad Prism 5 software (GraphPad Software, La Jolla, CA). Results in the figures are displayed as the mean ± SEM. Neutralizing antibody data was analyzed by nonlinear regression to obtain IC50 values, which were compared between immunization groups using ANOVA with a Dunnett’s multiple comparison test with Veh. serving as the control. Statistical significance was determined between sero- and sero+ groups within each immunization cohort using 2-way ANOVA with a Bonferroni post-test. P values less than 0.05 were considered significant.

## Results

### Prior RSV exposure and vaccine adjuvant influence IgG2a:IgG1 ratio and viral protection

**Figure 1:**
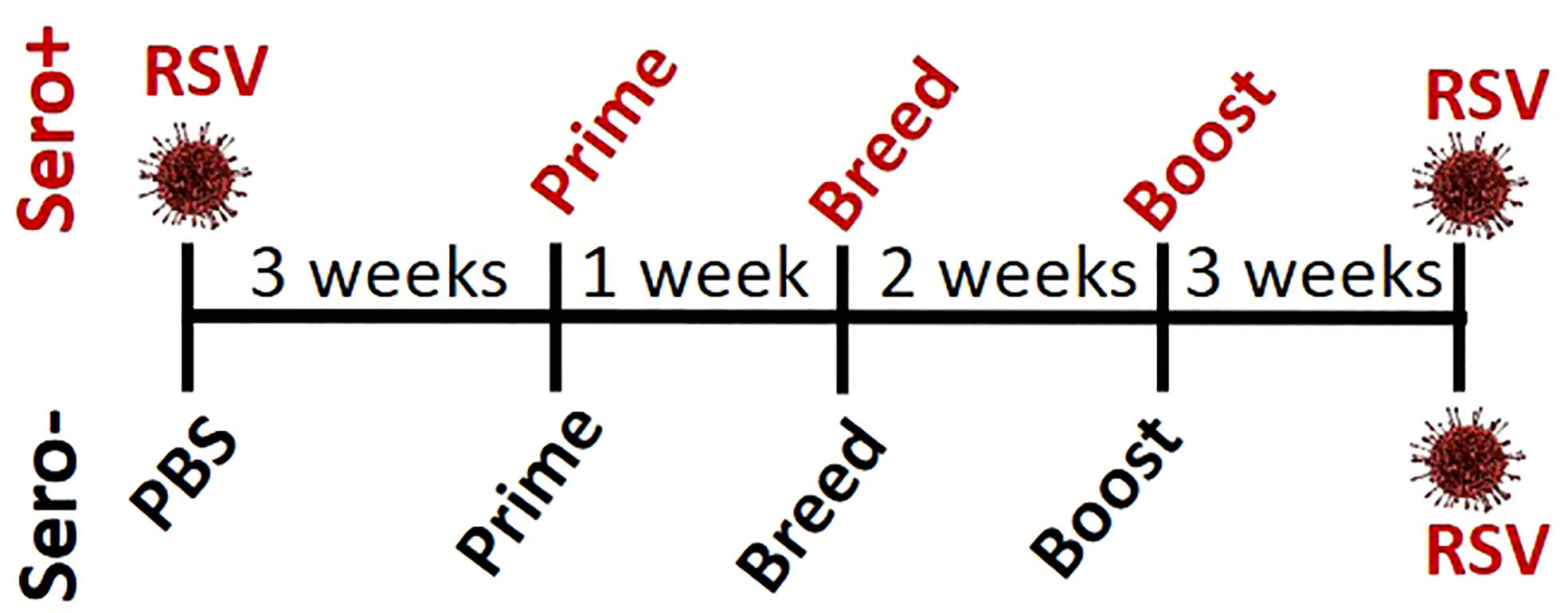
Study design.

To determine if RSV exposure prior to immunization alters the IgG2a:IgG1 ratio or neutralizing antibody titers, 4-week-old BALB/cJ mice were infected with RSV (sero+) and allowed to recover for 3 weeks before receiving an intramuscular immunization with: 1) Vehicle (Veh), 2) PreF alone (PreF), 3) PreF plus the Th-balanced adjuvant, Advax-SM (PreFAdx), 4) PreF plus the Th2 adjuvant, Alum (PreFAlum). The same immunization groups were created in age-matched mice with no prior RSV exposure (sero-). One week after prime, mice were bred with naïve, unimmunized male BALB/cJ mice for timed delivery of offspring. Three weeks after prime, dams received a secondary immunization of their respective vaccine formulations. PreF specific IgG subtypes and neutralization titers (NT) were measured from pre-challenge serum to assess immune responses generated by immunization. The dams were challenged with RSV 5 weeks after their final immunization according to the experimental schematic shown in **Figure 1**.

**Figure 2:**
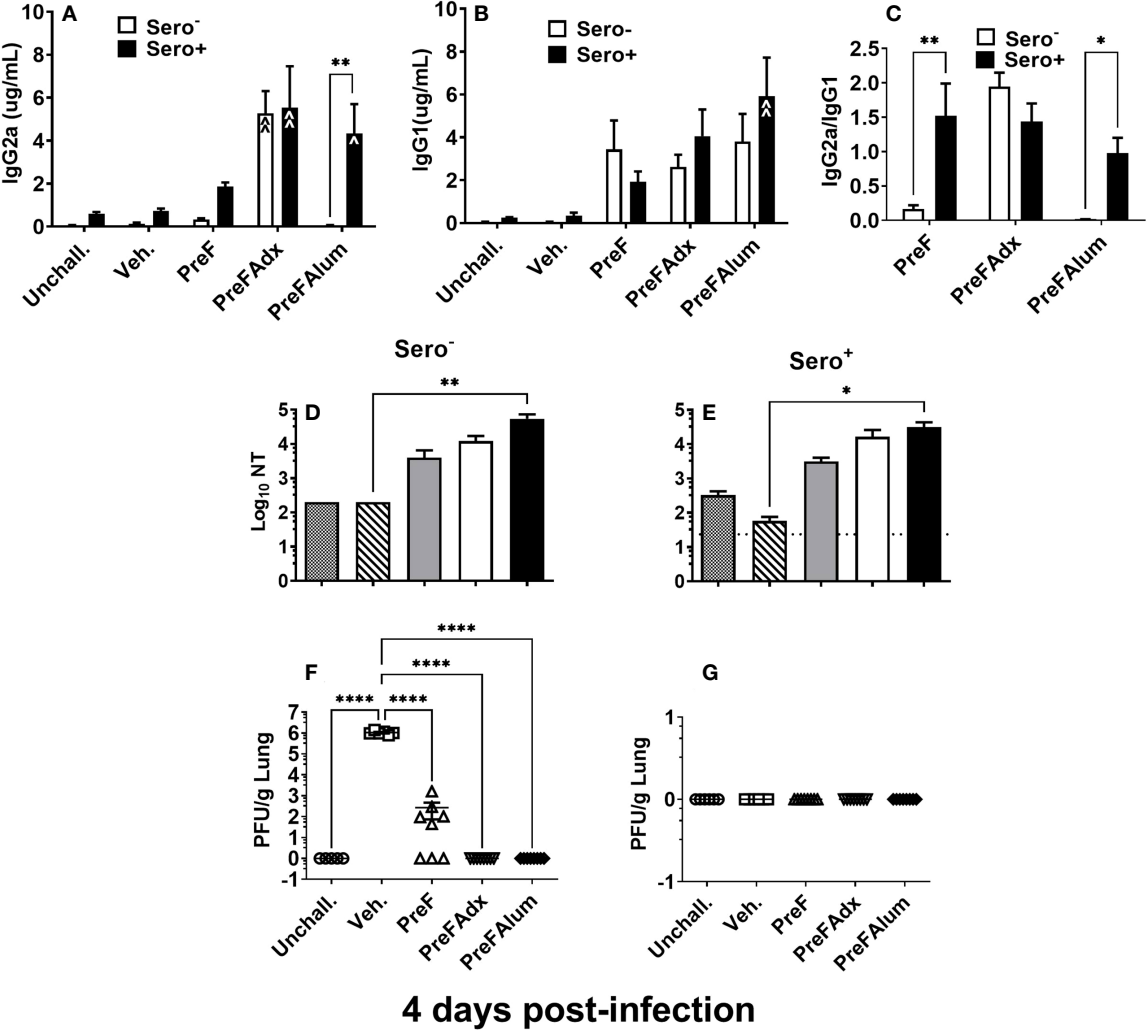
Prior RSV exposure increases the IgG2a/IgG1 ratio in immunized mice. Sero- and sero+ mice were immunized and challenged with RSV as described in **Figure 1**. RSV-specific IgG2a **(A)**, IgG1 **(B)**, the IgG2a/IgG1 ratio **(C)**, and Log10 NTs **(D, E)** were determined from pre-challenge serum collected immediately prior to RSV challenge. At 4 dpi, dams were culled, and tissues collected for analysis. Left lungs were harvested and processed for viral quantification using H&E plaque assays **(F, G)**. Viral titers were performed in triplicate and data within each group represent the mean titer for each animal. Lungs with no detectable virus were considered sterile. Data are represented as mean ± SEM of two independent experiments (*n* = 8 mice). Statistical significance was determined between sero- and sero+ groups within each immunization cohort using 2-way ANOVA with a Bonferroni post-test; *p<0.05, **p<0.01. Nonlinear regression was used to obtain IC50 values **(D, E)** and comparisons to Veh controls within the respective sero- and sero+ groups were made using an ANOVA with a Dunnett’s multiple comparison test; **(A, B)** ^p<0.05, ^^p<0.01 and **(D–G)** *p<0.05, **p<0.01, ****p<0.0001.

Sero- dams immunized with PreFAdx, but not those immunized with PreFAlum, generated higher titers of PreF-specific IgG2a compared to vehicle controls, indicating a T helper type 1 (Th1) response, in sero- mice immunized with PreFAdvax, but not PreFAlum (**Figure 2A**). In contrast, IgG2a was increased sero+ mice that were immunized with either PreFAdx or PreFAlum compared to vehicle controls (**Figure 2A**). PreF-specific IgG1 titers, indicative of a Th2-type response, trended higher in sero- and sero+ PreF, PreFAdx, and PreFAlum dams compared to their respective vehicle controls, but IgG1 was significantly higher only in sero+ dams immunized with PreFAlum (**Figure 2B**). No differences in IgG1 titers were detected between sero- and sero+ dams in any immunization groups tested (**Figure 2B**). Evaluation of IgG2a:IgG1 ratios from immunized dams revealed the ratio was below 0.5 in sero- PreF and PreFAlum dams, suggesting that immunization with unadjuvanted or alum-adjuvanted PreF without prior RSV infection favors a Th2 dominant response following viral challenge (**Figure 2C**). Alternatively, immunization of sero- dams with PreFAdx increased the IgG2a:IgG1 ratio to approximately 2, suggesting a predominant Th1 response following viral challenge in sero- dams. Alternatively, previous RSV exposure significantly increased the IgG2a:IgG1 ratio in sero+ dams immunized with PreF and PreFAlum (**Figure 2C**). Together, these data suggest that vaccine-mediated Th2 skewing, as determined by an IgG2a:IgG1ratio <1, is reduced in sero+ dams.

Evaluation of neutralizing antibody titers showed that PreF, PreFAdx, and PreFAlum dams all had increased neutralization titers (NTs) compared to Veh but only PreFAlum dams had significantly higher NTs and was independent of serostatus (**Figures 2D, E**). While Log_10_ NT appeared similar between sero- and sero+ dams, the assays between groups were run on different plates and could not be statistically compared (**Figures 2D, E**). Neutralizing antibody curves, as measured by RSV-Renilla Luciferase virus (**Figures S1-2**) reflect the neutralizing titers shown in **Figures 2D, E**.

Following RSV challenge, viral titers were quantified using H&E plaque assays. In sero- dams, all PreFAdx (n=8/8) and PreFAlum (n=8/8) dams achieved sterilizing immunity at 4 days post-infection (dpi) (**Figure 2F**), whereas only 37.5% (n=3/8) of dams immunized with PreF alone demonstrated sterilizing immunity (**Figure 2F**). Prior RSV exposure protected all sero+ dams from RSV challenge as indicated by complete sterilizing immunity in all experimental groups following viral challenge (**Figure 2G**). Taken together, these data suggest that serostatus and the type of adjuvant used are important predictors of RSV protection and IgG2a:IgG1 ratio, respectively, in BALB/c dams.

### Prior RSV exposure attenuates Th1- and Th2-dominant T cell responses to PreFAdx and PreFAlum immunization, respectively, and increases the RSV F-specific CD8 T cell response

**Figure 3:**
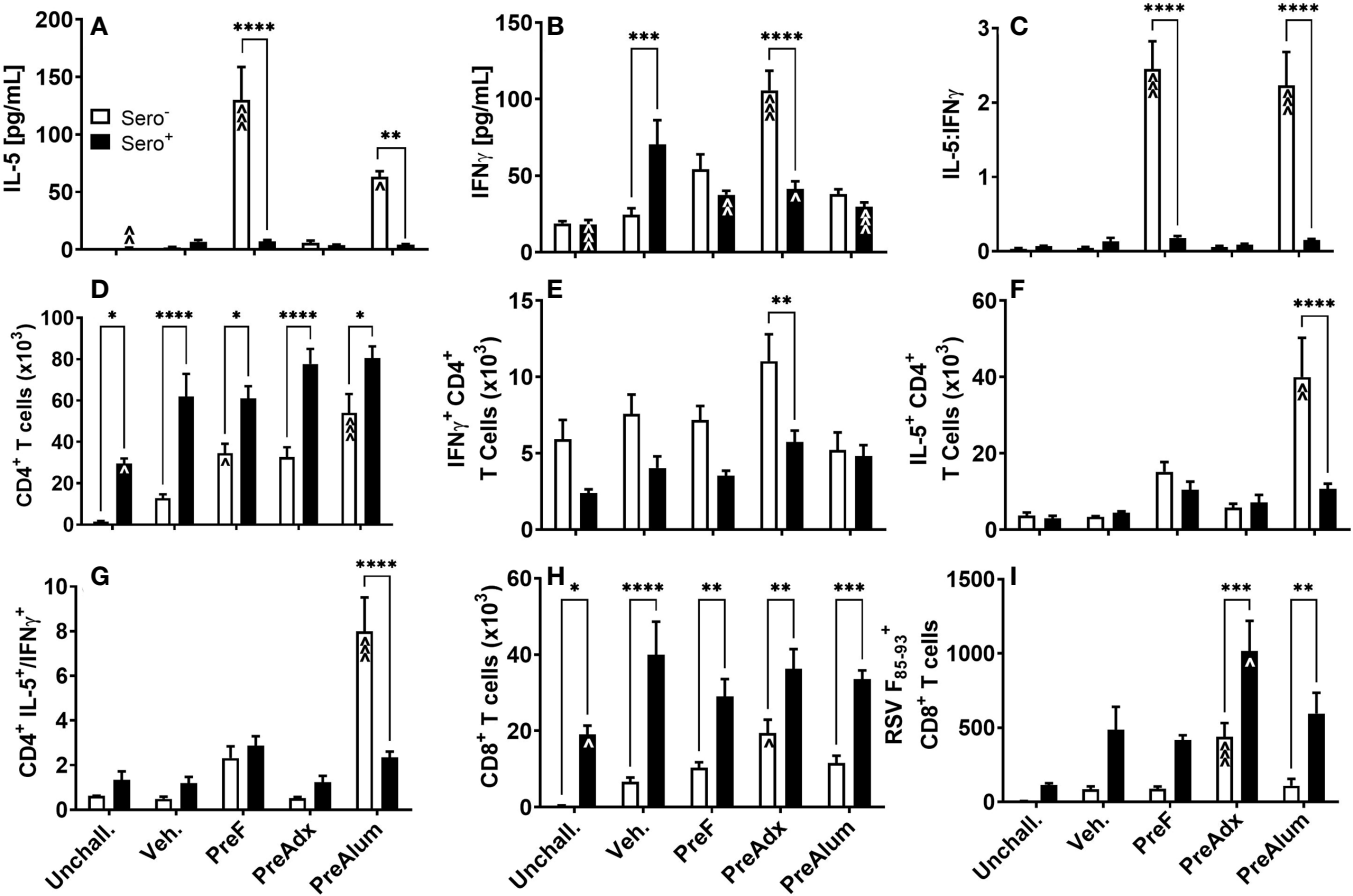
Prior RSV exposure mitigates Th1- and 2-type responses linked to vaccination in sero- mice but increases RSV F_85-93_-specific CD8 T cells. Sero- and sero+ mice were immunized and challenged with virus as described in **Figure 1**. At 4dpi, IL-5 **(A)** and IFNγ **(B)** were quantified in the BAL by Luminex and the ratio of IL-5/IFNγ **(C)** was calculated. Total CD4 **(D)** and CD8 **(H)** T cells as well as RSV F_85-93_-specific CD8 T cells **(I)** were measured in the BAL by flow cytometry. Intracellular production of IFNγ **(E)** and IL-5 **(F)** by CD4 T cells was quantified in BAL and the ratio of IL-5/IFNγ produced by CD4 T cells was calculated **(G)**. Data are represented as mean ± SEM of two independent experiments (*n* = 8 mice). Statistical significance was determined between sero- and sero+ groups within each immunization cohort using 2-way ANOVA with a Bonferroni post-test; *p<0.05, **p<0.01, ***p<0.001, and ****p<0.0001. Comparisons to Veh controls within the respective sero- and sero+ groups were made using an ANOVA with a Dunnett’s multiple comparison test; ^p<0.05, ^^p<0.01, and ^^^p<0.001.

To determine the extent to which serostatus influenced the Th1- and Th2-type response to vaccination, cytokines were quantified in the BAL of sero- and sero+ immunized dams following RSV challenge. Consistent with the IgG2a:IgG1 ratios shown in **Figure 2C**, concentrations of the canonical Th2 cytokine, IL-5, were elevated in sero- PreF and PreFAlum dams compared to the sero- vehicle control at the 4-day timepoint (**Figure 3A**), though it should be pointed out that responses in the vehicle group may increase over time. Compared to the sero- mice, IL-5 was significantly reduced in the respective sero+ immunization groups and was not increased relative to the sero+ vehicle control mice (**Figure 3A**). The Th1 cytokine, IFNγ, was increased in sero- PreFAdx dams relative to vehicle control, whereas concentrations were significantly lower in sero+ PreFAdx dams (**Figure 3B**). In the sero+ cohort, IFNγ levels were greater in the Veh. group compared to all the other immunization groups and the unchallenged controls, suggesting that immunization of sero+ dams reduced the production of IFNγ elicited by secondary viral challenge (**Figure 3B**). The IL-5:IFNγ ratio was greater than 1 in sero- PreF and PreFAlum dams indicating an overall Th2 dominant immunization response following RSV challenge compared to vehicle control (**Figure 3C**). Whereas, RSV exposure prior to vaccination dramatically reduced the IL-5:IFNγ ratio in these two immunization groups to values less than 0.5, suggesting that prior RSV exposure reduces Th1- and Th2-type cytokine responses.

To determine the extent to which IL-5 and IFNγ were being produced by CD4 T cells, BAL was harvested, and intracellular cytokine staining was performed. The CD4^+^ T cell populations were increased in sero+ dams, regardless of immunization strategy, compared to the respective unchallenged mice (**Figure 3D**). Alternatively, in the sero- cohort, only dams immunized with PreF or PreFAlum had higher levels of CD4 T cells compared to their respective sero- vehicle control (**Figure 3D**). CD4 T cell populations were increased in all sero+ compared to sero- groups, suggesting that prior RSV exposure was driving the increase in CD4 T cells. Interestingly, CD4^+^ T cells were also elevated in the sero+ unchallenged group, which did not receive a secondary RSV challenge, indicating a residual CD4^+^ T cell population in the airspace 11 weeks after initial RSV exposure and suggests that CD4 T cells will continue to increase over time in the sero- cohort (**Figure 3D**). Alternatively, the number of CD4^+^ T cells producing IFNγ in the lungs of sero+ dams trended down compared to sero- dams in all groups except PreFAlum and only PreFAdx dams were significantly different (**Figure 3E**). Despite the large increase in soluble IL-5 in sero- PreF and PreFAlum groups (**Figure 3A**), only sero- PreFAlum, but not PreF immunized dams had an increase in IL-5-producing CD4^+^ T cells in the BAL with a parallel increased ratio of IL-5 to IFNγ producing CD4^+^ T cells (**Figures 3F, G**), suggesting a possible alternate cellular source of IL-5 in the PreF group. Like CD4^+^ T cells, CD8^+^ T cells were more frequent in the airways of sero+ compared to sero- immunized dams, with the largest relative increase observed in CD8^+^ T cells from unimmunized Veh. dams (**Figure 3H**). Among immunized sero- dams, only the PreFAdx group had an increase in CD8^+^ T cells compared to the respective vehicle control (**Figure 3H**). Using an MHCI RSV F_85-93_ protein pentamer, we showed that immunization with PreFAdx increased the number of RSV F-specific CD8 T cells in both sero- and sero+ dams compared to their respective vehicle controls. However, sero+ dams had markedly increased RSV F-specific CD8 T cells in both PreFAdx and PreFAlum groups compared to their respective sero- counterparts (**Figure 3I**). Together, these data show that PreFAdx elicited a Th1-biased response in BALB/c dams whereas PreFAlum and PreF alone elicited a Th2-biased response in sero- dams, that was largely mitigated in sero+ dams. Moreover, these data show that prior RSV exposure increased T cell responses regardless of immunization regimen and enhanced the RSV F_85-93_-specific CD8^+^ T cell response in PreFAdx- and PreFAlum-immunized dams compared to previous RSV exposure alone (Veh.).

### Th2-cytokine producing ILC2s are associated with increased lung pathology in sero^-^ PreF and PreFAlum dams

**Figure 4:**
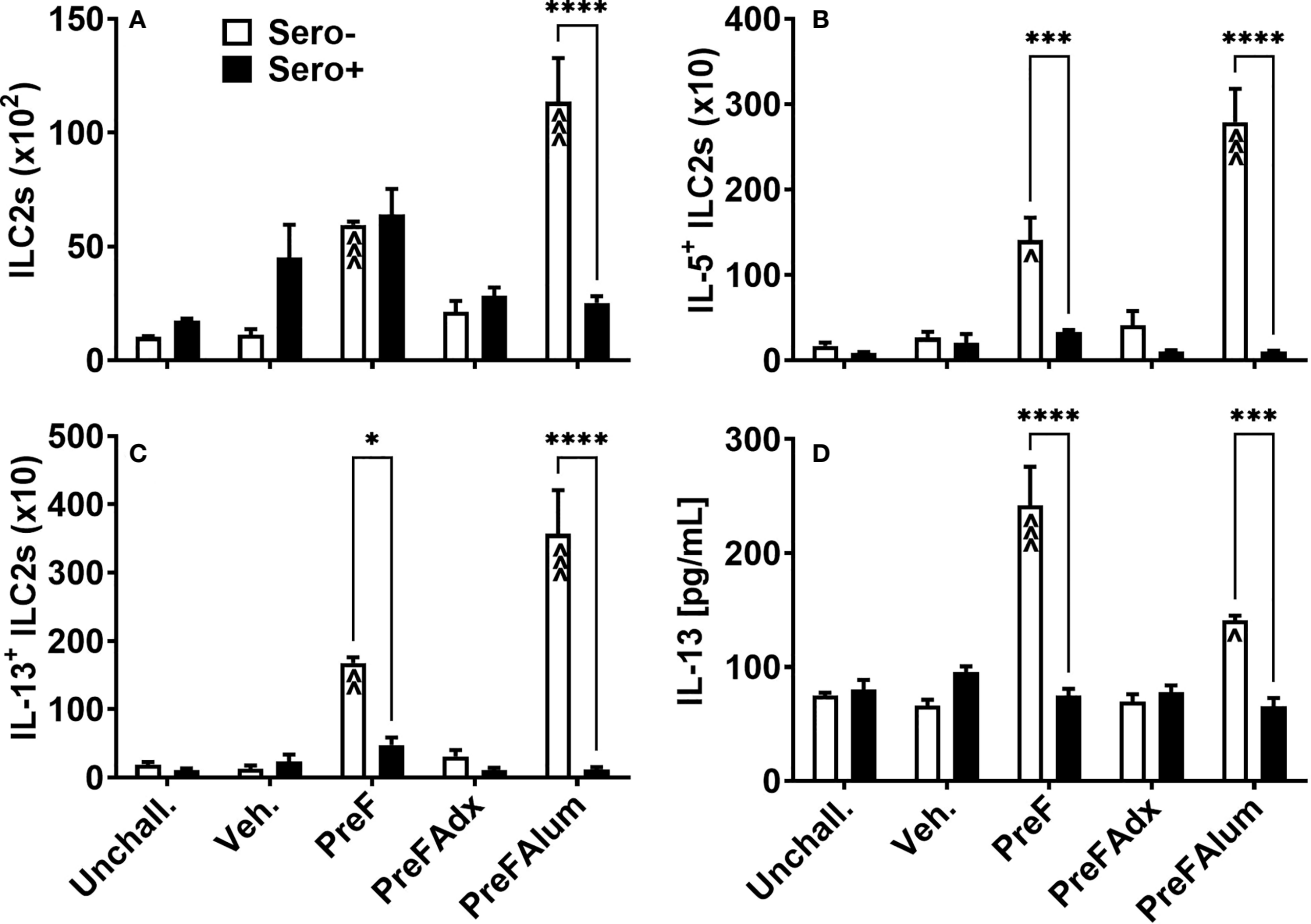
Th2-polarizing vaccine regimens are associated with increased ILC2 activation in sero- but not sero+ mice. Sero- and sero+ mice were immunized and challenged with virus as described in **Figure 1**. At 4dpi, lungs were harvested for quantification of ILC2s **(A)**, IL-5+ILC2s **(B)**, and IL-13+ ILC2s **(C)** by flow cytometry. IL-13 was measured in the BAL by Luminex **(D)**. Data are represented as mean ± SEM of two independent experiments (*n* = 8 mice). Statistical significance was determined between sero- and sero+ groups within each immunization cohort using 2-way ANOVA with a Bonferroni post-test; *p<0.05, ***p<0.001, and ****p<0.0001. Comparisons to Veh controls within the respective sero- and sero+ groups were made using an ANOVA with a Dunnett’s multiple comparison test; ^p<0.05, ^^p<0.01, and ^^^p<0.001.

Based on their known contribution to the Th2-type cytokine milieu in the lung, namely IL-5 and IL-13, we sought to determine if ILC2s were a contributing source of IL-5 in the PreF group and if prior RSV exposure influenced ILC2 production of IL-5 and IL-13. At 4 dpi, sero- PreF and PreFAlum dams had elevated numbers of ILC2s (Lin^-^ CD45^+^ ST2^+^ IL7Rα^+^) compared to their sero- vehicle control (**Figure 4A**). Compared to sero+ dams, sero- PreFAlum dams had significantly greater ILC2 **(** **Figure 4A**). Moreover, ILC2-producing IL-5 (**Figure 4B**) and IL-13 (**Figure 4C**) were markedly increased in PreF- and PreFAlum-immunized sero- compared to sero+ dams (**Figures 4B, C**), suggesting that prior infection mitigated the ILC2 response following RSV challenge. The increase in IL-5- and IL-13-producing ILC2s paralleled increases in soluble IL-5 (**Figure 3A**) and IL-13 (**Figure 4D**) measured in the BAL of sero- PreF and PreFAlum dams. Importantly, IL-5- and IL-13-producing ILC2s were not increased in dams immunized with PreFAdx relative to vehicle controls in sero- or sero+ groups. Taken together, these data show that ILC2s contribute to IL-5 and IL-13 production in sero- PreF- and PreFAlum-immunized dams and that prior exposure to RSV mitigated the ILC2 response.

### Airway eosinophils and neutrophils are differentially increased in sero^-^ and sero^+^ dams

**Figure 5:**
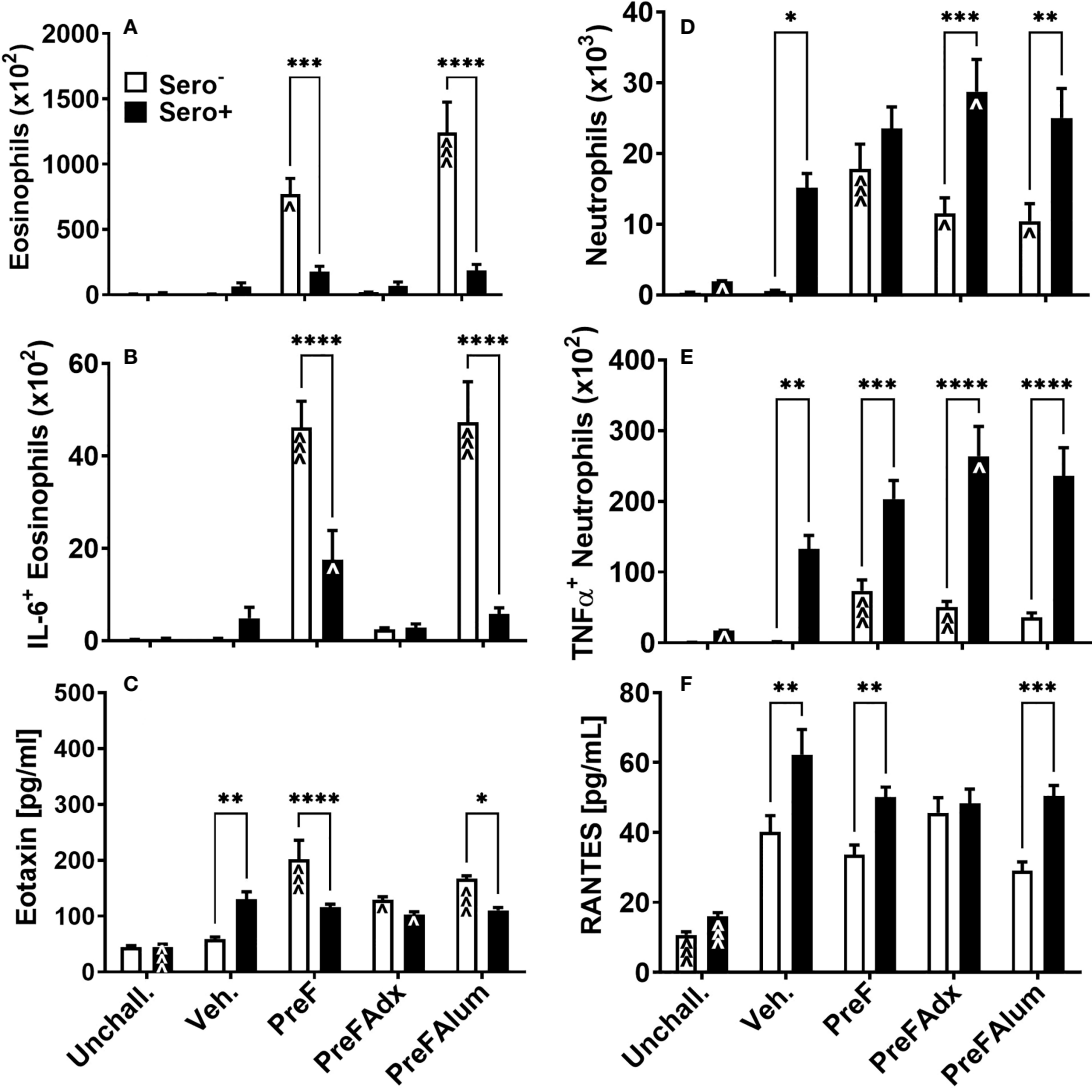
Eosinophils and neutrophils are differentially increased in immunized sero- and sero+ mice. Sero- and sero+ mice were immunized and challenged with virus as described in **Figure 1**. At 4dpi, BAL was harvested for quantification of eosinophils **(A)**, IL-6+ eosinophils **(B)**, neutrophils **(D)**, and TNFα+ neutrophils **(E)**. Soluble eotaxin **(C)** and RANTES **(F)** were determined in the BAL by Luminex. Data are represented as mean ± SEM of two independent experiments (*n* = 8 mice). Statistical significance was determined between sero- and sero+ groups within each immunization cohort using 2-way ANOVA with a Bonferroni post-test; *p<0.05, **p<0.01, ***p<0.001, and ****p<0.0001. Comparisons to Veh. controls within the respective sero- and sero+ groups were made using an ANOVA with a Dunnett’s multiple comparison test; ^p<0.05, ^^p<0.01, and ^^^p<0.001.

Based on the increase in ILC2- and CD4 T cell-derived IL-5 production, a known recruiter and activator of eosinophils, we sought to determine if PreF- and PreFAlum-immunized dams had elevated eosinophils in their airways and whether this might be mitigated by prior RSV exposure. At 4 dpi, total eosinophils in the BAL of immunized dams were highest in sero- PreF and PreFAlum dams compared to sero- vehicle control and their respective sero+ immunization groups (**Figure 5A**). In sero- PreF and PreFAlum dams, airway eosinophils produced high levels of IL-6 (**Figure 5B**), a cytokine involved in the acute phase response and Th2 differentiation (23). Similar to the reduction seen in Th2-type cytokine-secreting ILC2 populations in sero+ PreF and PreFAlum dams, total airway eosinophils and IL-6^+^ eosinophils were significantly reduced in sero+ compared to sero- PreF and PreFAlum dams, though IL-6^+^ eosinophils were still increased in the PreF sero+ immunization group relative to their vehicle control (**Figure 5B**). Increases in eotaxin, a potent eosinophil chemokine, paralleled that of eosinophils in the sero- PreF and PreFAlum groups relative to their vehicle control and sero+ counterparts (**Figure 5C**). Despite increases in eotaxin in both sero- and sero+ PreFAdx dams relative to their vehicle controls, no parallel increase in eosinophils was observed in these groups at the 4-day time point (**Figure 5C**).

In contrast to eosinophils, which were most prominent in sero- PreF and PreFAlum groups, neutrophils, another signature inflammatory cell recruited during vaccine-associated ERD, was increased in all immunized sero- dams (PreF, PreFAdx, and PreFAlum) relative to their vehicle control (**Figure 5D**). Alternatively, neutrophils in all sero+ groups (Veh, PreF, PreFAdx, and PreFAlum) were increased over sero+ unchallenged dams (**Figure 5C**), suggesting that repeat RSV challenge rather than vaccination was driving the lung neutrophil responses in sero+ dams at 4dpi. Increases in TNFα- producing neutrophils increased in concert with the increase in total neutrophils among sero- and sero+ dams within each immunization group (**Figure 5E**) and was more pronounced in sero+ dams. RANTES, a potent chemokine involved in neutrophil recruitment, was elevated in sero+ compared to sero- dams, with the exception of PreFAdx-immunized dams (**Figure 5F**) and may thereby have contributed to the increase in neutrophil recruitment. Taken together, these data suggest that the Th2 vaccine regimen (PreFAlum) drove an eosinophil response in sero- dams, whereas prior infection drove neutrophil responses in sero+ dams independent of the vaccine regimen.

### Prior RSV exposure does not protect against lung pathology in immunized BALB/c dams

**Figure 6:**
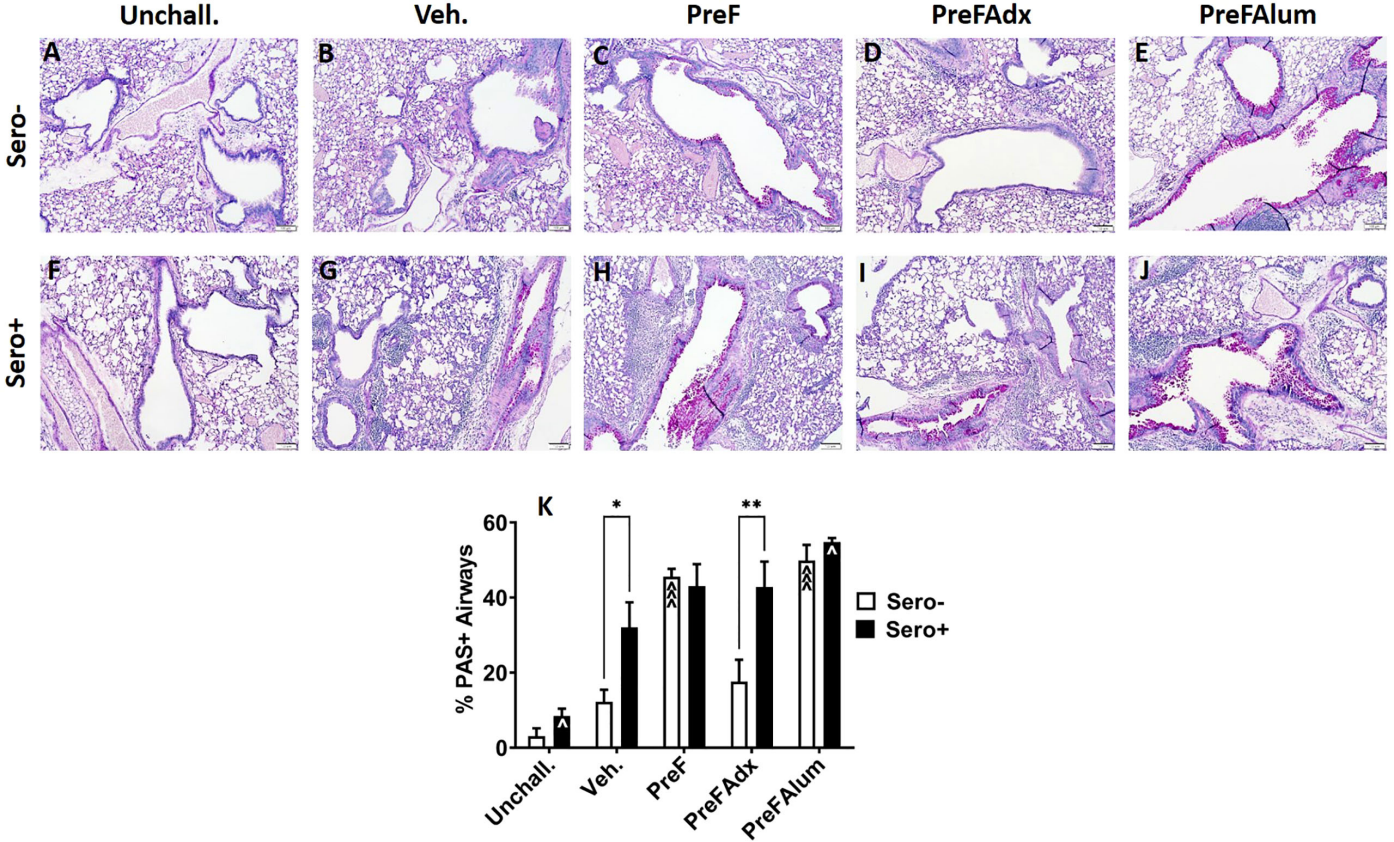
PAS staining is increased in sero- PreF- and PreFAlum-immunized mice and in sero+ mice independent of immunization regimen. Sero- and sero+ mice were immunized and challenged with virus as described in **Figure 1**. At 4dpi, sero- **(A–E)** and sero+ **(F–J)** lungs were formalin filled, paraffin embedded and sectioned for staining with PAS. Two independent, blinded investigators scored all slides as described in the methods. Scores between the two investigators were averaged and data represented as mean ± SEM (*n* = 3-4 mice). Statistical significance was determined between sero- and sero+ groups within each immunization cohort using 2-way ANOVA with a Bonferroni post-test **(K)**; *p<0.05 and **p<0.01. Comparisons to Veh. controls within the respective sero- and sero+ groups were made using an ANOVA with a Dunnett’s multiple comparison test; ^p<0.05 and ^^^p<0.001.

Based on the Th2 bias and eosinophilic response in sero- PreF and PreFAlum-immunized dams, we asked if lung pathology was also increased in these groups and if prior RSV infection mitigated this pathology. Sero- dams immunized with PreF or PreFAlum had increased airway mucus production at 4 dpi as evidenced by abundant PAS staining compared to vehicle controls (**Figures 6A–C, E, K**). Conversely, sero- PreFAdx dams (**Figure 6D**) were protected from excess mucus production and had a low percentage of PAS+ airways (**Figure 6K**) that was on par with Veh. controls (**Figures 6D, K**). In the sero+ dams, Veh. controls had increased PAS staining by 4dpi compared to unchallenged mice (**Figures 6F, G, K**). Moreover, sero+ vehicle controls had increased PAS staining over sero- vehicle controls which received a primary RSV challenge only (**Figures 6B, G, K**). Notably, sero+ dams immunized with PreFAlum had a significant increase in the percentage of PAS+ airways compared to Veh. controls (**Figures 6G, J, K**). In contrast to the sero- cohort, sero+ dams immunized with PreFAdx (**Figure 6I**) developed extensive PAS staining but was not greater than the sero+ vehicle control (**Figures 6D, I, K**). Sero+ dams immunized with PreF alone (**Figure 6H**) maintained similar levels of PAS+ airways as compared to their sero- counterparts (**Figure 6K**). A more detailed analysis of the PAS severity scores confirmed that mucus production (scores 2-4) was elevated among sero- PreF- and PreFAlum- immunized groups compared to vehicle control, but not in sero- dams immunized with PreFAdx (24). In the sero+ cohort an increased PAS score (between 2-4) was seen in all 4 groups after secondary RSV challenge (sero+) relative to mice that remained unchallenged (**Figures S3**). Taken together, these data show that although the %PAS+ airways are greater following repeat RSV challenge (likely due to a faster and more robust immune response) compared to primary RSV challenge (sero-), only mice in the PreFAlum group had increased PAS scores compared to vehicle controls.

**Figure 7:**
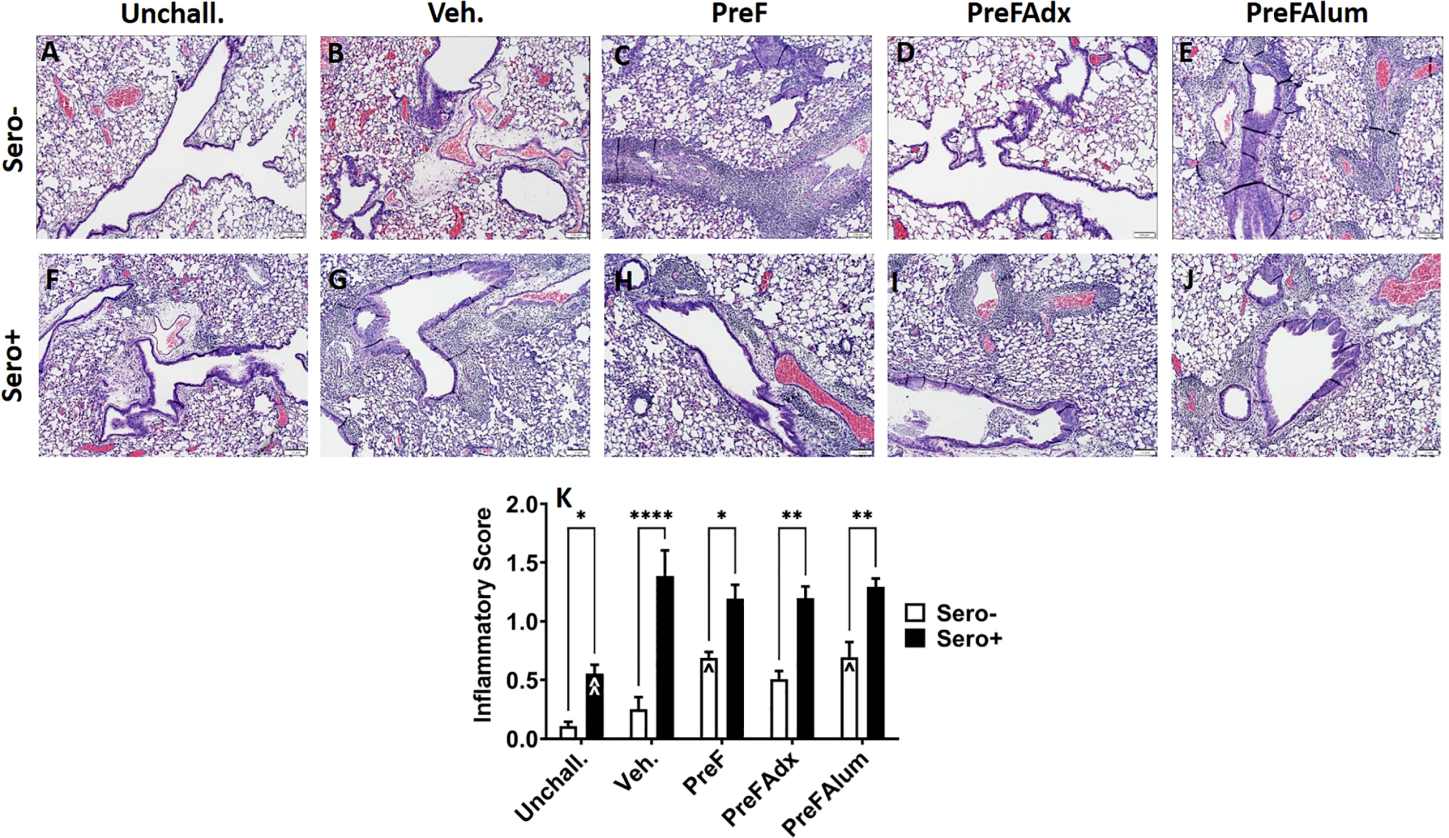
H&E staining is increased in sero- PreF- and PreFAlum-immunized mice and in sero+ mice independent of immunization regimen. Sero- and sero+ mice were immunized and challenged with virus as described in **Figure 1**. At 4dpi, sero- **(A–E)** and sero+ **(F–J)** lungs were formalin filled, paraffin embedded and sectioned for staining with H&E. Two independent pathologists, blinded to immunization group and serostatus, scored all slides as described in the methods. Scores between the two investigators were averaged and data represented as mean ± SEM (*n* = 3-4 mice). Statistical significance was determined between sero- and sero+ groups within each immunization cohort using 2-way ANOVA with a Bonferroni post-test **(K)**; *p<0.05, **p<0.01, and ****p>0.0001. Comparisons to Veh. controls within the respective sero- and sero+ groups were made using an ANOVA with a Dunnett’s multiple comparison test; ^p<0.05 and ^^p<0.01.

Similar to PAS staining, inflammation (as determined by H&E-stained lung sections) was increased in sero- dams immunized with PreF or PreFAlum when compared to vehicle controls at 4dpi. Alternatively, inflammation was not increased in sero- dams immunized with PreFAdx (**Figures 7A–E, K**). Inflammation was greater in all the sero+ compared sero- groups (**Figures 7A–K**), likely due to a more rapid memory response to repeat RSV challenge. Sero+ Veh. dams (**Figure 7G**) showed the largest increase in inflammation over the sero- dams (**Figure 7B**), although this was largely due to the low degree of inflammation in sero- Veh. dams at the 4-day timepoint (**Figure 4K**). When average scores were graphed as a percentage of the total number of scored fields of view (**Figures S4, 5**), sero- dams that were immunized with PreF, PreFAdx, or PreFAlum had an increased frequency of inflammatory scores of 1 or 2 compared to vehicle. However, PreF and PreFAlum dams had higher overall percentages of these scores compared to PreFAdx dams. Surprisingly, sero+ Unchall. dams still had mild inflammation approximately 11 weeks after their initial RSV exposure. Together, these data suggest that a Th2 vaccine regimen is associated with increased mucus production and inflammation after challenge in the lungs of sero- dams, which is mitigated by a more Th1 biased vaccine regimen, PreFAdx. Alternatively, in the previously exposed sero+ cohort, only PreFAlum showed increased mucus production compared to vehicle controls and inflammation was not increased over vehicle controls.

## Discussion

Because nearly all women of child-bearing age have been previously infected with RSV, maternal immunization strategies are thought to provide a safe and effective means by which to protect vulnerable children during their first months of life. Moreover, protection of children born to immunized mothers is short lived, so direct immunization strategies in seropositive children may serve as an additional approach to protect vulnerable populations (Clinicaltrials.gov; NCT03387137). Despite the marked differences in host immune response between naïve and RSV-experienced individuals, most preclinical vaccine models test safety and efficacy in naïve animals only (10, 25–32). Thus, the studies reported here evaluated the capacity of Th2-polarizing versus Th1-biased vaccine regimens, administered prior to and during pregnancy, to protect dams from subsequent RSV infection and lung pathology in both sero- and sero+ mice.

Consistent with previously reported vaccine studies in sero- mice, Th2-skewed immunity, lung inflammation, and mucus production was increased in mice immunized with prefusion RSV F antigen (PreF) alone or with PreFAlum (24, 33). While mice in both these immunization groups had Th2-mediated lung pathology following RSV challenge, mice immunized with PreF alone had incomplete viral protection, whereas mice in the PreFAlum group had undetectable virus, suggesting that active viral replication is not required for enhanced disease in mice. In contrast to the FI-RSV clinical trials (3), in which poorly neutralizing antibody and immune complex deposition was implicated in enhanced disease (34), antibody generated from the stabilized PreF antigen in these studies was potently neutralizing. While immune complex deposition was not measured in the current studies, results suggest that low antibody levels generated from the PreF alone group and the Th2-skewing activity of alum in the PreFAlum group, contributed to the observed Th2-type inflammation and mucus production. Neutralizing antibody levels were high in the PreFAlum groups, suggesting that poorly neutralizing antibody was not a cause of pathology in this group. Thus, in addition to poorly neutralizing antibodies (34), results of these studies are consistent with those showing that low antibody titers (35, 36) and alum adjuvant (24, 37) also contribute to lung inflammation, but do so by driving differential cellular responses.

Results of these studies further show that vaccine-mediated Th2-skewing by PreFAlum and the Th1-skewing by PreFAdx observed in sero- dams was largely mitigated in sero+ dams, suggesting that prior RSV exposure reduces vaccine-mediated skewing. This is in stark contrast to sero- dams in which immunization with the Th2-polarizing alum-adjuvanted PreF vaccine elicited a dominant Th2 CD4 T cell response, increased IL-13- and IL-5-producing ILC2 and eosinophils with associated mucus production and peribronchial and perivascular lung inflammation. Moreover, sero- dams immunized with PreFAdx (a non-Th2 -polarizing regimen), had negligible type II cytokines (IL-5 and IL-13) and increased IFNγ levels. This Th1-biased response was associated with an absence of inflammation and mucus production following RSV challenge, suggesting that a Th1-promoting adjuvant may prevent development of ERD under sero- conditions. Consistent with these findings, a recent report showed that immunization with an alum-adjuvanted RSV F protein was associated with Th2-type inflammation and mucus production in RSV naïve mice but was prevented when RSV F protein was formulated with monophosphoryl lipid A (MPL) or CpG oligonucleotide, adjuvants (30). These data further align with previous reports describing protection against ERD when the Th1 cytokine, IFNγ, was delivered at the time of primary infant RSV infection, either by inhalation (18, 38) or recombinant virus (39). Also, in contrast to the Th2-polarzing immunization groups, mice in the PreFAdx group had a significant increase in RSV F_85-93_ specific CD8 T cells, which paralleled the increase in soluble IFNγ in the BAL. This data is consistent with our previously published work showing that intranasal delivery of recombinant murine IFNγ at the time of RSV infection increased RSV-F_85-93_-specific CD8 T cell responses (40).

The skewed Th1 and Th2 responses observed in sero- mice immunized with PreFAdx and PreFAlum, respectively, were absent in sero+ dams. Despite increased in inflammation and mucus production in sero+ mice relative to unchallenged controls at 4dpi, neither type II cytokines (IL-5 and IL-13) nor the type I cytokine IFNγ were increased in response to immunization in with PreFAlum or PreFAdx, respectively. Importantly, the increase in RSV F_85-93+_ CD8 T cells was observed in both sero+ and sero- dams compared to their respective vehicle controls and warrants further studies to determine if vaccine strategies that promote RSV-specific memory CD8 T cell responses may provide better, more long-term protection in all populations regardless of serostatus.

In the current studies, an unexpected but striking difference in immune response was the complete abrogation of IL-5- and IL-13-producing ILC2 in sero+ dams compared to PreFAlum- and PreF- immunized sero- dams at the 4-day time point. Sero- dams that were immunized with PreFAlum or PreF alone had a marked increase in IL-5- and IL-13-producing ILC2 in lung homogenate compared to vehicle controls. When exposed to RSV prior to immunization with PreFAlum or PreF, IL-5- and IL-13-producing ILC2 were completely mitigated. The role of IL-13-producing ILC2 was previously reported in a mouse model of RSV infection where thymic stromal lymphopoietin (TSLP) or IL-33 released from infected airway epithelium triggered ILC2 activation and production of IL-13 (41, 42). The marked increase in IL-5- and IL-13-producing ILC2s in sero- PreFAlum mice is particularly intriguing considering no replicating virus was detected at 4dpi. Moreover, ILC2 activation was greater in sero- mice immunized with PreFAlum, which imparted complete viral protection at 4dpi, compared to sero- veh. controls. Together, these data suggest there may be additional mechanisms of ILC2 activation that extend beyond viral damage to the airway epithelium.

Our data showed a correlative increase in Th2-type cytokine-producing CD4 T cells and ILC2s in the sero- PreF and PreFAlum groups. Alternatively, while CD4 T cells were increased in sero+ mice, cytokine-producing (IL-5- and IFNγ) CD4 T cells and ILCs were not increased. This data is consistent with a recent report showing that ILC2s are not effectively activated during RSV infection when cytokine-producing CD4 T cells are depleted (43). Moreover, in a mouse model of Nippostrongylus brasiliensis, ILC2 and T cell crosstalk were shown to contribute to their mutual maintenance, expansion, and cytokine production (44). In our studies total ILC2 producing IL-5 and IL-13 were reduced in sero+ compared to sero- dams in correlation with a reduction in IL-5-producing CD4 T cells in the BAL and lung homogenate. As IL-5- and IL-13-producing ILC2 were completely blocked in PreFAdx-immunized sero- mice relative to vehicle controls, it is conceivable, that vaccine-mediated generation of Th1- versus Th2- CD4 memory T cells provide additional signals or crosstalk to mitigate ILC2 activation. To this point, MHCII-mediated dialogue between ILC2 and CD4 T cells have been reported in helminth infections (44), and may play an important role in vaccine-mediated responses. Previously published reports in murine models of RSV infection and human studies have implicated ILC2s in RSV-mediated pathology. However, to our knowledge, this is the first report linking ILC2s with vaccine associated ERD and may serve as a potential cellular target for vaccine development.

Taken together, our data suggest that vaccine-mediated Th-skewing is mitigated in sero+ mice. This is consistent with anecdotal reports that there are no known cases of vaccine associated ERD reported in seropositive children (3, 5, 6, 45, 46), and suggests that prior infection protects against vaccine-mediated Th2-type lung inflammation. Alternatively, it is also well appreciated that early life RSV infection does not protect against subsequent RSV disease (47–49). Our data is also consistent with these findings, whereby a uniformly high level of inflammation and mucus production was observed at the early 4 day timepoint in sero+ vehicle control dams relative to unchallenged controls, which was not observed in sero- dams. This is likely attributed to a delayed, rather than a lack of, inflammation in sero- vehicle control mice following primary RSV infection. Consistent with this idea, we previously reported that neutrophils, lymphocytes, and monocytes increase in parallel with increased inflammation following primary RSV infection in sero- mice at a later 8 day timepoint (24). In the current study, inflammation in the sero+ dams was similarly driven by neutrophils, lymphocytes and monocytes (**Figure S6**), suggesting that the response to RSV infection is expedited in sero+ compared to sero- mice. Further work is needed to understand the mechanism by which prior infection buffers the Th1- or Th2- driven response.

In summary, results of these studies illustrate the importance of differential immune responses to RSV immunization based on vaccine regimen (Th1 vs Th2 bias) and history of RSV infection and highlight the need to incorporate sero+ animals in preclinical vaccine safety studies. Future studies will be needed to determine if RSV vaccine regimens that favor Th1 type immunity can overcome the Th2-biased immune response established by RSV infection and if preimmunity differentially protects infants born to immunized mothers.

## Data availability statement

The raw data supporting the conclusions of this article will be made available by the authors, without undue reservation.

## Ethics statement

The animal study was reviewed and approved by The University of Pittsburgh Institutional Animal Care and Use Committee.

## Author contributions

KEi, JK, and KEm contributed to the overall study design, execution, and interpretation of data and writing of the paper. MY contributed to data generation, study design, and analysis. TP and TO contributed to the analysis and interpretation of data. CM and NP contributed by contributing test agents and review of data. All authors contributed to the article and approved the submitted version.

## Funding

Financial support was provided by 1R43AI140941-01 (PI: MY/KEm), David and Betty Brenneman Fund (PI: KEm), 5T32AI089443-07 (KEi/PI: Shlomchik), 1TL1TR001858 (KEi/PI: Kapoor), Cystic Fibrosis Foundation Research Grant – OURY19GO (PI: TO), R03-RHD080874A (PI: KEm). This work benefitted from SPECIAL BD LSRFORTESSATM funded by NIH 1S10OD011925-01 (PI: Borghesi). NP (Vaxine Pty Ltd., Bedford Park, Australia) generously provided Advax-SM but did not otherwise provide financial assistance for this project. Development of Advax-SM ajuvant was supported in part by from National Institute of Allergy and Infectious Diseases of the National Institutes of Health under Contracts HHS-N272201400053C, HHS-N272200800039C and U01-AI061142. This publication’s contents are solely the responsibility of the authors and do not necessarily represent the official views of the National Institutes of Health, National Institute of Allergy and Infectious Diseases.

## Conflict of interest

NP is affiliated with Vaxine Pty Ltd., which hold commercial interests in Advax adjuvants. CM and MY are affiliated with Calder Biosciences, which holds commercial interests in the stabilized PreF protein.

The remaining authors declare that the research was conducted in the absence of any commercial or financial relationships that could be construed as a potential conflict of interest.

## Publisher’s note

All claims expressed in this article are solely those of the authors and do not necessarily represent those of their affiliated organizations, or those of the publisher, the editors and the reviewers. Any product that may be evaluated in this article, or claim that may be made by its manufacturer, is not guaranteed or endorsed by the publisher.
