# Supplementary material for: Prior respiratory syncytial virus infection reduces vaccine-mediated Th2-skewed immunity, but retains enhanced RSV F-specific CD8 T cell responses elicited by a Th1-skewing vaccine formulation
Source: Front Immunol. 2022 Oct 4;13:1025341. doi: 10.3389/fimmu.2022.1025341 (PMC9577258; doi:10.3389/fimmu.2022.1025341)

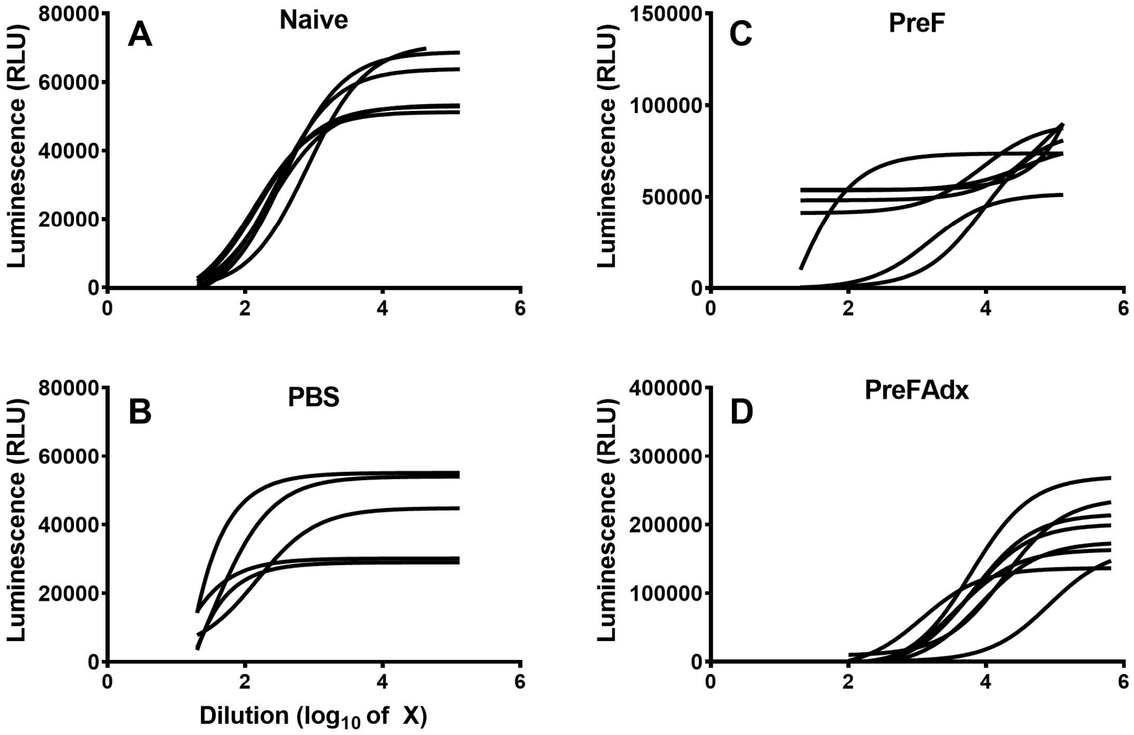

Figure S2. In seropositive mice, viral luminescence is markedly reduced in PreFAdx and PreFAlum groups. Pre-challenge serum was collected via submandibular bleed 2-3 days prior to RSV challenge in seronegative mice immunized with Naïve (A), PBS (B), PreF (C), PreFAlum (D), and PreFAdx (E). Serial dilutions of heat inactivated serum were incubated in a 96-well plate format with 100 pfu/well Line 19 RSV-Renilla Luciferase virus (provided by the Moore laboratory). Luciferase activity (luminescence) was measured using a Novostar plate reader after a 15-minute incubation at 25ºC. All plates were run in duplicate and averaged.

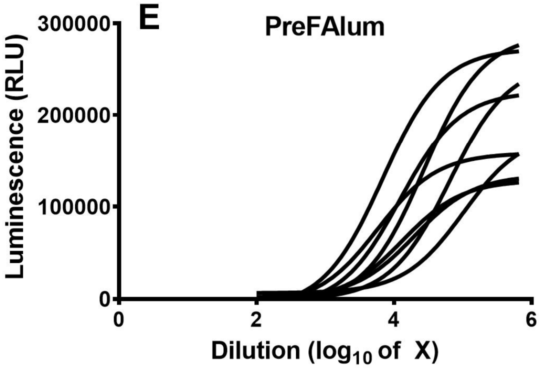

Supplement: Supplementary file 2 [file DataSheet_2.pdf]
