# Supplementary material for: Prior respiratory syncytial virus infection reduces vaccine-mediated Th2-skewed immunity, but retains enhanced RSV F-specific CD8 T cell responses elicited by a Th1-skewing vaccine formulation
Source: Front Immunol. 2022 Oct 4;13:1025341. doi: 10.3389/fimmu.2022.1025341 (PMC9577258; doi:10.3389/fimmu.2022.1025341)
Supplement: Supplementary file 3 [file DataSheet_3.pdf]

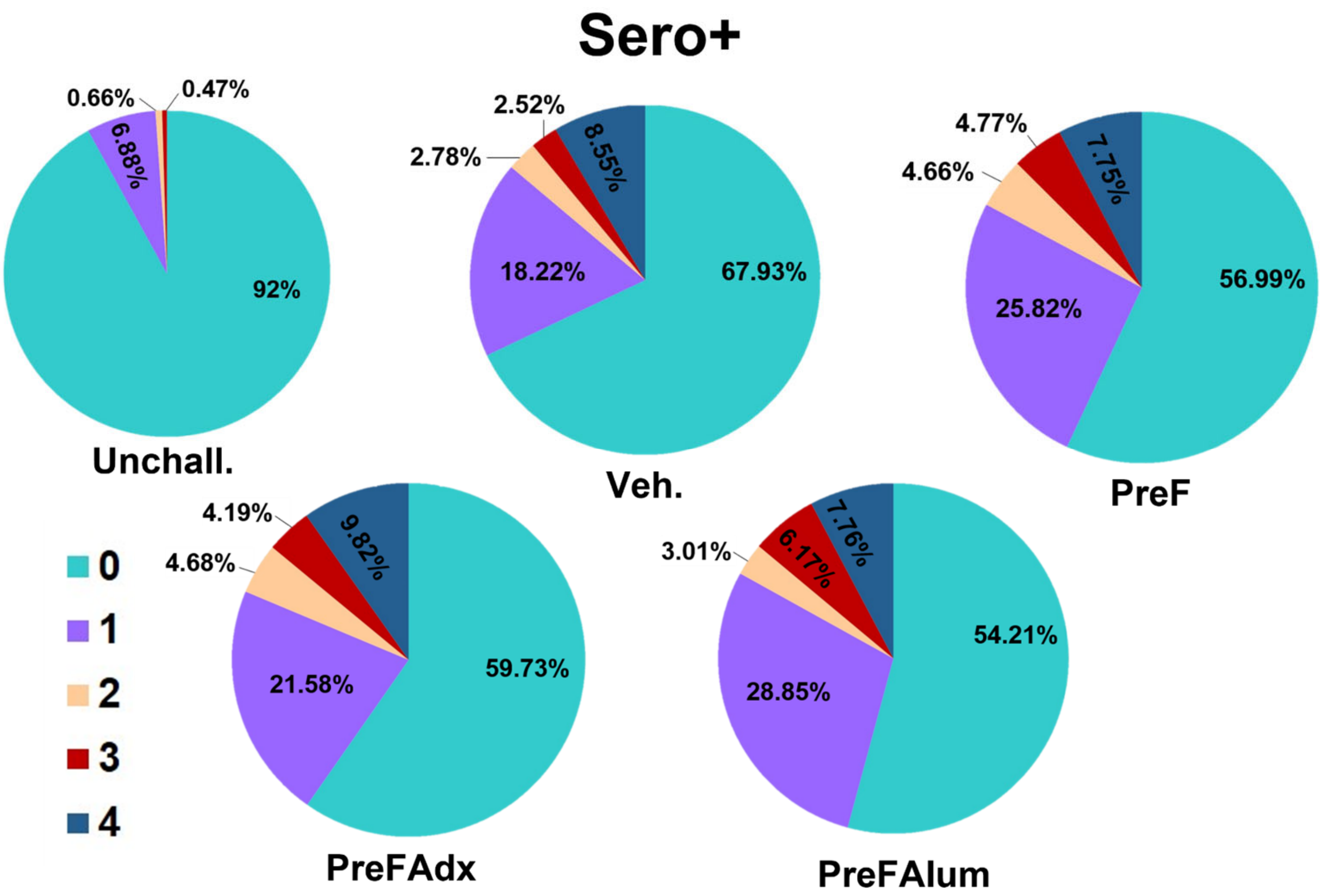

Figure S3. Breakdown of PAS scores in sero+ mice shows predominantly level 1 and 4 mucus production. Sero+ mice were immunized and challenged with virus as described in Figure 1. To quantify the extent of PAS staining, lungs were scored as previously described in the methods. Briefly, a score of 0 - 5 was given to all airways (average 55) with the following scale: 0 = no PAS+ cells; 1 = 1-25% PAS+ cells; 2 = 26-50% PAS+ cells; 3 = 51-75% PAS+ cells; 4 = 76-100% PAS+ cells. Scores were averaged and the total percentage of PAS+ airways were graphed along with a more detailed breakdown of the ratio of each severity score (0 – 5) divided by the total number of airways.
