# Supplementary material for: Prior respiratory syncytial virus infection reduces vaccine-mediated Th2-skewed immunity, but retains enhanced RSV F-specific CD8 T cell responses elicited by a Th1-skewing vaccine formulation
Source: Front Immunol. 2022 Oct 4;13:1025341. doi: 10.3389/fimmu.2022.1025341 (PMC9577258; doi:10.3389/fimmu.2022.1025341)
Supplement: Supplementary file 4 [file DataSheet_4.pdf]

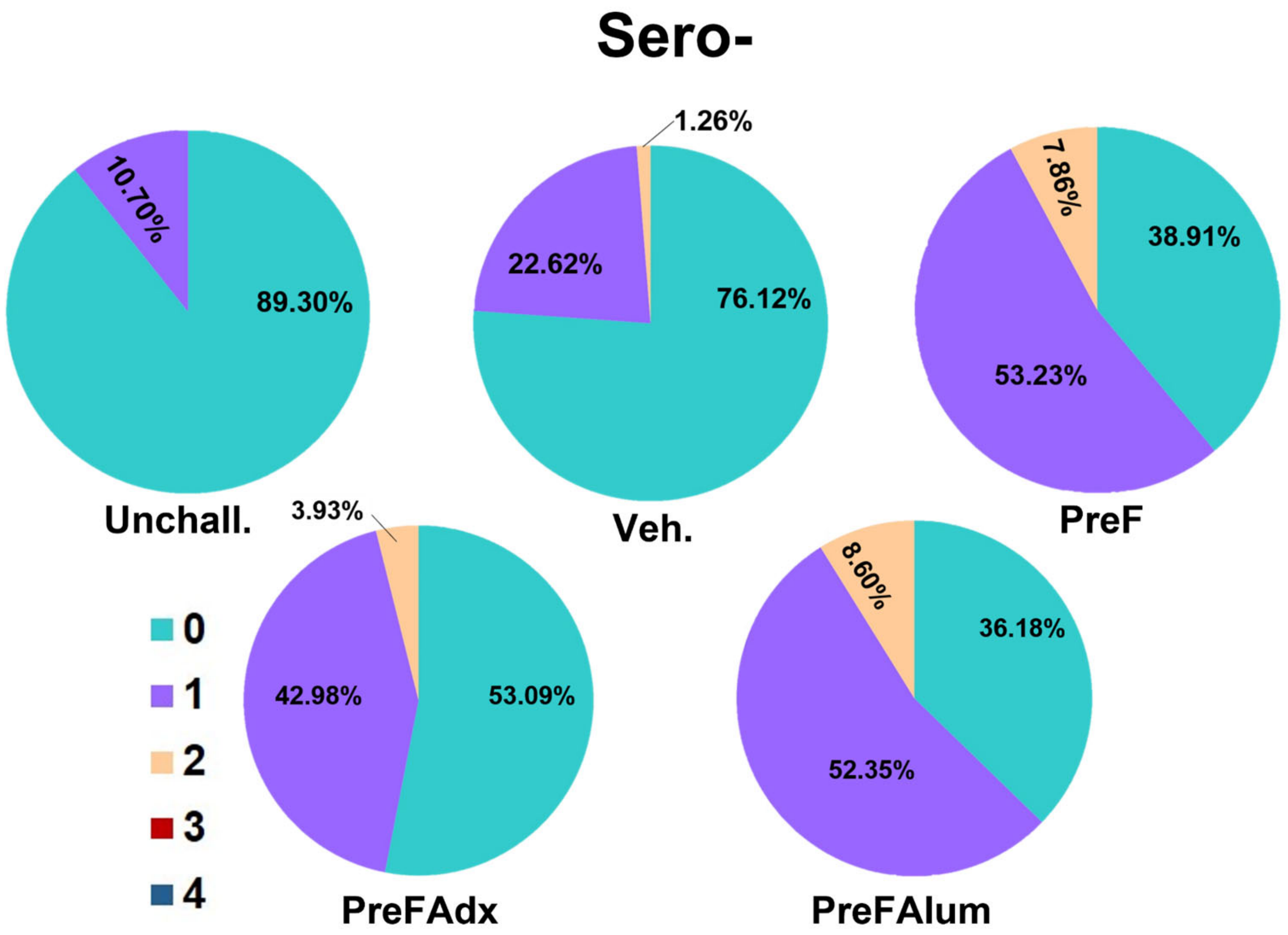

Figure S4. Breakdown of H&E scores in sero- mice shows predominantly level 1 inflammation. Sero- mice were immunized and challenged with virus as described in Figure 1. To quantify the extent of H&E staining, lungs were scored as described in the methods. Briefly, standard hematoxylin and eosin staining was performed on lung sections and scored by two pathologists blinded to treatment groups. In short, each field (average 28 fields) in the lung was observed with a light microscope (x200 magnification) and scoring was based on the percentage of lung tissue affected according to the following scale: 0 = no inflammation, 1 = up to 25%, 2 = 25 – 50%, 3 = 50 – 75%, and 4 = 75 – 100%. Scores were averaged and reported as a ratio of the sum of scores divided by the total number of fields counted. Severity scores were also graphed as a fraction of the total number of fields counted.
