# Supplementary material for: Prior respiratory syncytial virus infection reduces vaccine-mediated Th2-skewed immunity, but retains enhanced RSV F-specific CD8 T cell responses elicited by a Th1-skewing vaccine formulation
Source: Front Immunol. 2022 Oct 4;13:1025341. doi: 10.3389/fimmu.2022.1025341 (PMC9577258; doi:10.3389/fimmu.2022.1025341)
Supplement: Supplementary file 6 [file DataSheet_6.pdf]

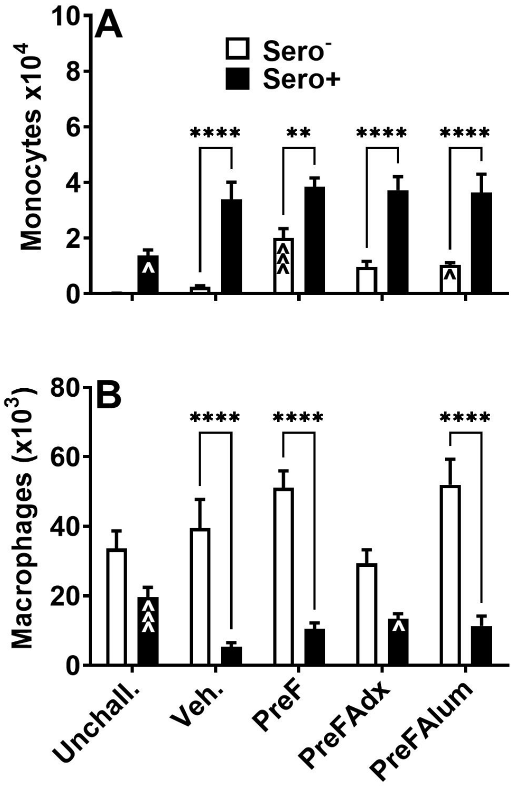

Figure S6. Monocytes were increased in sero+ monocytes independent of vaccine regimen whereas increased macrophages in sero- mice were reduced in PreFAdx-immunized mice . Sero- and sero+ mice were immunized and challenged with virus as described in Figure 1. At 4dpi, monocytes (A) and macrophages (B) were measured in the BAL by flow cytometry. Statistical significance was determined between sero- and sero+ groups within each immunization cohort using 2-way ANOVA with a Bonferroni post-test; *p<0.05, **p<0.01, and ****p>0.0001. Comparisons to Veh. controls within the respective sero- and sero+ groups were made using an ANOVA with a Dunnett's multiple comparison test; ^p<0.05 and ^^p<0.01.
